# Post-Prandial Protein Handling: You Are What You Just Ate

**DOI:** 10.1371/journal.pone.0141582

**Published:** 2015-11-10

**Authors:** Bart B. L. Groen, Astrid M. Horstman, Henrike M. Hamer, Michiel de Haan, Janneau van Kranenburg, Jörgen Bierau, Martijn Poeze, Will K. W. H. Wodzig, Blake B. Rasmussen, Luc J. C. van Loon

**Affiliations:** 1 Top Institute Food and Nutrition, Wageningen, The Netherlands; 2 Department of Human Movement Sciences, Maastricht University Medical Centre, Maastricht, The Netherlands; 3 Department of Radiology, Maastricht University Medical Centre, Maastricht, The Netherlands; 4 Laboratory Biochemical Genetics, Department of Clinical Genetics, Maastricht University Medical Centre, Maastricht, The Netherlands; 5 Department of Surgery, Maastricht University Medical Centre, Maastricht, The Netherlands; 6 Central Diagnostic Laboratory, Maastricht University Medical Centre, Maastricht, The Netherlands; 7 Department of Nutrition and Metabolism, University of Texas Medical Branch, Galveston, Texas, United States of America; University of Bath, UNITED KINGDOM

## Abstract

**Background:**

Protein turnover in skeletal muscle tissue is highly responsive to nutrient intake in healthy adults.

**Objective:**

To provide a comprehensive overview of post-prandial protein handling, ranging from dietary protein digestion and amino acid absorption, the uptake of dietary protein derived amino acids over the leg, the post-prandial stimulation of muscle protein synthesis rates, to the incorporation of dietary protein derived amino acids in *de novo* muscle protein.

**Design:**

12 healthy young males ingested 20 g intrinsically [1-^13^C]-phenylalanine labeled protein. In addition, primed continuous L-[ring-^2^H_5_]-phenylalanine, L-[ring-^2^H_2_]-tyrosine, and L-[1-^13^C]-leucine infusions were applied, with frequent collection of arterial and venous blood samples, and muscle biopsies throughout a 5 h post-prandial period. Dietary protein digestion, amino acid absorption, splanchnic amino acid extraction, amino acid uptake over the leg, and subsequent muscle protein synthesis were measured within a single *in vivo* human experiment.

**Results:**

55.3±2.7% of the protein-derived phenylalanine was released in the circulation during the 5 h post-prandial period. The post-prandial rise in plasma essential amino acid availability improved leg muscle protein balance (from -291±72 to 103±66 μM·min^-1^·100 mL leg volume^-1^; *P*<0.001). Muscle protein synthesis rates increased significantly following protein ingestion (0.029±0.002 vs 0.044±0.004%·h^-1^ based upon the muscle protein bound L-[ring-^2^H_5_]-phenylalanine enrichments (*P*<0.01)), with substantial incorporation of dietary protein derived L-[1-^13^C]-phenylalanine into *de novo* muscle protein (from 0 to 0.0201±0.0025 MPE).

**Conclusion:**

Ingestion of a single meal-like amount of protein allows ~55% of the protein derived amino acids to become available in the circulation, thereby improving whole-body and leg protein balance. About 20% of the dietary protein derived amino acids released in the circulation are taken up in skeletal muscle tissue following protein ingestion, thereby stimulating muscle protein synthesis rates and providing precursors for *de novo* muscle protein synthesis.

**Trial Registration:**

trialregister.nl 3638

## Introduction

It has been well established that protein turnover in skeletal muscle tissue is highly responsive to nutrient intake in healthy adults. Protein ingestion increases both muscle protein synthesis as well as muscle protein breakdown rates, albeit the latter to a lesser extent, resulting in a positive net muscle protein balance [1–3]. The post-prandial muscle protein synthetic and anti-proteolytic response to feeding is regulated on various levels, ranging from protein digestion and amino acid absorption [4, 5], the post-prandial rise in circulating insulin and subsequent increase in microvascular recruitment [6, 7], amino acid uptake in skeletal muscle tissue [8], intramuscular anabolic signaling [9, 10] and myofibrillar muscle protein synthesis and breakdown [11]. It has been proven difficult to define the impact of all of these variables on the anabolic response to protein feeding in an *in vivo* human setting.

To investigate dietary protein digestion and amino acid absorption kinetics, we have previously combined the ingestion of specifically produced intrinsically L-[1-^13^C]-phenylalanine labeled milk protein [12] with the continuous intravenous infusion of L-[ring-^2^H_5_]- phenylalanine [5, 13–22]. The use of intrinsically labeled protein allows us to determine the rate at which dietary protein derived amino acids enter the circulation and provides us with an estimate of splanchnic amino acid retention [21]. Others have applied the AV-model [23] to assess post-prandial amino acid uptake and release by measuring arterio-venous differences in plasma amino acid concentrations over the leg combined with the assessment of leg blood flow [24–26]. Post-prandial stimulation of muscle protein synthesis is typically assessed by the measurement of the increase in muscle protein bound L-[ring-^2^H_5_]-phenylalanine following a primed continuous infusion of L-[ring-^2^H_5_]-phenylalanine with the plasma or muscle free L-[ring-^2^H_5_]-phenylalanine being used as precursor pool [4, 27, 28]. To overcome the methodological limitations associated with the non-steady state and precursor pool dilution when assessing post-prandial muscle protein synthesis, Reitelseder *et al*. [29] introduced the assessment of post-prandial muscle protein synthesis rates by the primed continuous infusion of L-[1-^13^C]-leucine combined with the ingestion of intrinsically L-[1-^13^C]-leucine labeled protein (~5–10 MPE), thereby preventing a post-prandial decline in plasma precursor pool enrichment. By the production of intrinsically labeled protein with high L-[1-^13^C]-phenylalanine enrichment levels (~30–40 MPE), we have been able to assess the metabolic fate of dietary protein derived phenylalanine by directly measuring post-prandial L-[1-^13^C]-phenylalanine incorporation in muscle protein [21, 30, 31]. Recently, we introduced the production and application of L-[1-^13^C]-leucine (~10 MPE) and L-[1-^13^C]-phenylalanine (~35 MPE) labeled milk proteins [32]. Combining the ingestion of intrinsically L-[1-^13^C]-leucine and L-[1-^13^C]-phenylalanine labeled protein with intravenous L-[1-^13^C]-leucine and L-[ring-^2^H_5_]-phenylalanine infusion allows the assessment of dietary protein digestion and amino acid absorption kinetics, splanchnic extraction of protein derived amino acids, muscle protein synthesis rates, as well as the metabolic fate of dietary protein derived amino acids for *de novo* muscle protein synthesis [33].

We hypothesized that a substantial part of the ingested protein derived amino acids are directly used as amino acid precursors to support the post-prandial rise in *de novo* muscle protein synthesis rate. By applying a combination of methods we assessed *in vivo* dietary protein digestion and subsequent amino acid absorption, the appearance of dietary protein derived amino acids in the circulation, the uptake and release of amino acids over the leg, the post-prandial stimulation of muscle protein synthesis as well as the use of protein derived amino acids for *de novo* muscle protein synthesis within one single experiment. Twelve healthy males were selected to participate in this study in which these parameters were assessed prior to and after ingesting 20 g intrinsically labeled casein. Intravenous amino acid tracer infusions were applied with venous and arterial blood and muscle biopsy samples being collected frequently. This study quantifies post-prandial protein handling and the subsequent use of dietary protein derived amino acids for *de novo* muscle protein synthesis *in vivo* in humans. Consequently, this work provides a complete and comprehensive insight into the fact that you actually are what you just ate.

## Methods

### Subjects

**Fig 1:**
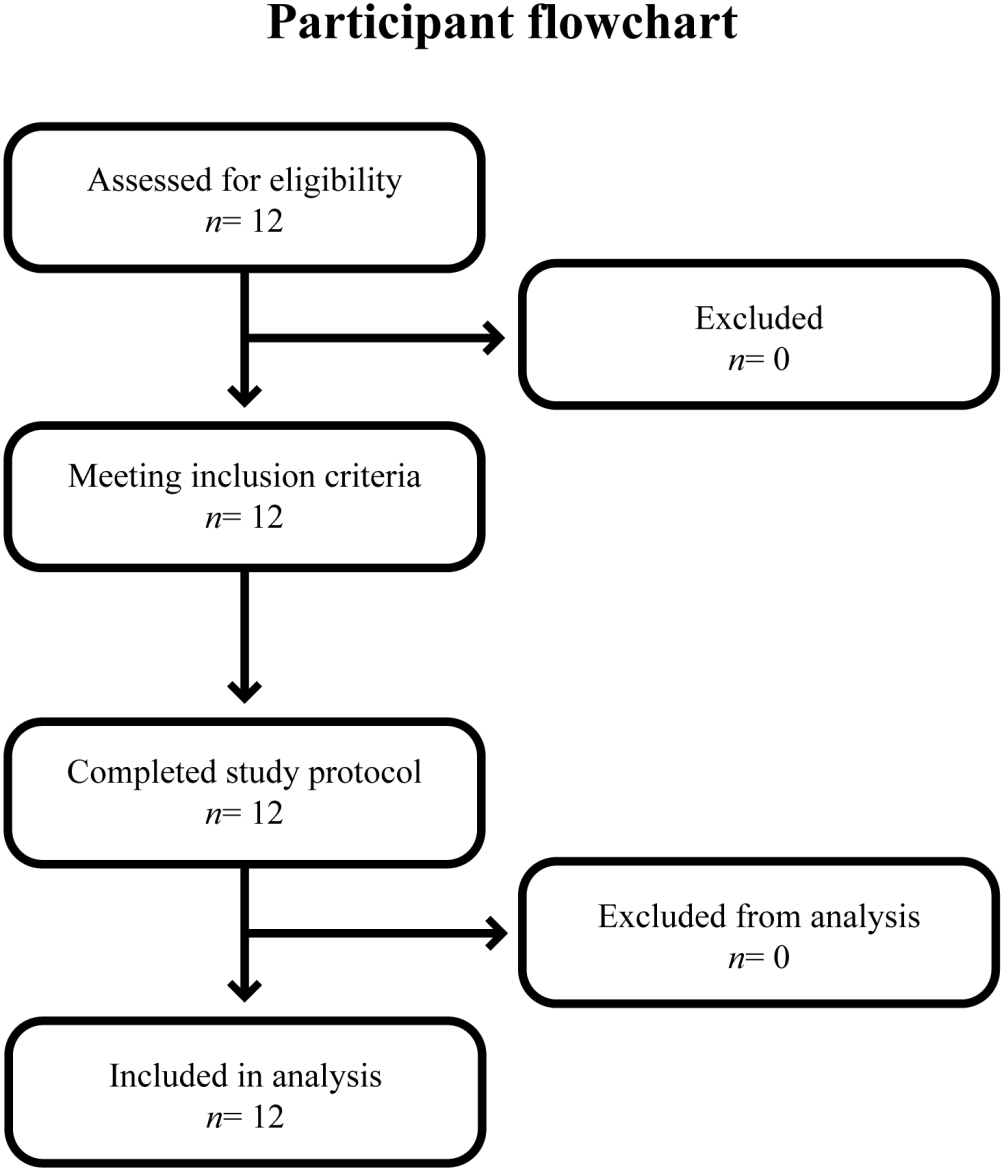
Flowchart of participants.

Twelve healthy, young males (age: 23±1 y; BMI: 22.6±0.3 kg·m^-2^) were recruited, pretested and selected to participate in the present study between March and November 2012 (Fig 1). Subjects’ characteristics are presented in Table 1. The power calculation was based upon a desired 10% increase of the fractional synthetic rate (FSR) from the post-absorptive to the post-prandial state following the ingestion of 20 g of protein [18]. The effect size we were looking to detect is 1.16, with an α = 0.05 and a power of 0.90, the *a priori* power calculation resulted in a total sample size of 12 subjects. All subjects were informed of the nature and possible risks of the experimental procedures, before their written informed consent was obtained. This study was approved by the Medical Ethical Committee of the Maastricht University Medical Centre and conforms to the principles outlined in the declaration of Helsinki for use of human subjects and tissue.

**Table 1:** Subjects’ characteristics.

	Subjects (*n* = 12)
Age (y)	23±1
Weight (kg)	72.5±1.8
BMI (kg)	22.6±0.3
HbA1c (%)	5.2±0.1
Fasting glucose (mmol·L^-1^)	4.7±0.2
OGIS	489±19
Blood pressure systolic (mmHg)	123±2
Blood pressure diastolic (mmHg)	63±2
Leg volume (L)	9.2±0.4
Leg mass (kg)	10.1±0.3
Appendicular lean mass (kg)	27.0±0.9
Lean body mass (kg)	58.2±1.7

Values are expressed as means ± SEM. HbA1c, glycated hemoglobin; OGIS, Oral Glucose Insulin Sensitivity.

### Pretesting

Following an overnight fast, participants arrived at the laboratory at 8:30 AM by car or public transport. Blood pressure was measured after which a baseline blood sample was collected to determine blood count, plasma electrolytes, liver (ALAT) and renal (creatinine and ureum) function, blood HbA1c levels, and plasma glucose concentrations. Subsequently, an oral glucose tolerance test was performed to determine oral glucose intolerance and/or the presence of type 2 diabetes according to American Diabetes Association Guidelines [34]. OGIS values were based on plasma glucose and insulin concentrations from the 2 h oral glucose tolerance test. Calculations were used as described by Mari *et al*. [35]. After glucose tolerance testing, leg volume was assessed as described by Jones and Pearson [36] with circumferences and segmental leg lengths taken at the gluteal furrow, mid-thigh, above the knee, maximum knee, below the knee, as well as maximum calf and minimum ankle circumferences [36]. Body composition was measured with DXA (Lunar Prodigy Advance; GE Health Care, Madison, WI). The system’s software package (en-CORE 2005, version 9.15.00) was used to determine whole body and regional lean mass, fat mass and bone mineral content. Healthy subjects with screening results within normal limits and stable body weight for at least 3 months were included.

### Study design

**Fig 2:**
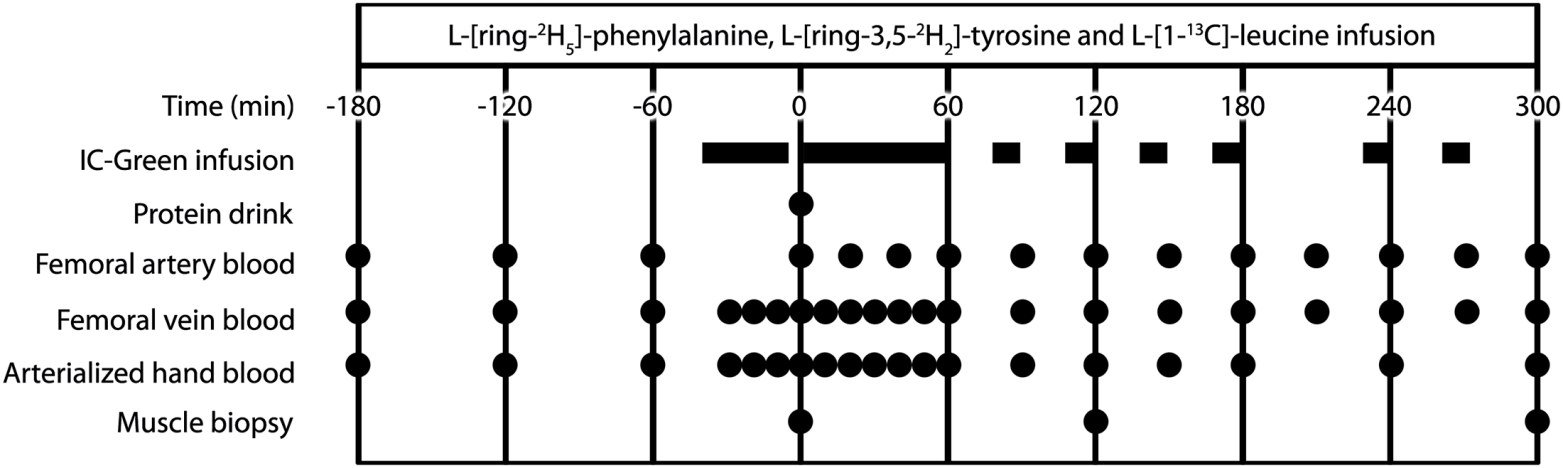
Outline of study protocol.

The experimental protocol is outlined in Fig 2. Each subject participated in one experiment, in which post-prandial protein handling was studied after the ingestion of 20 g intrinsically L-[1-^13^C]- phenylalanine and L-[1-^13^C]-leucine labeled protein. Antecubital venous and femoral arterial and venous lines were inserted [37] for intravenous L-[1-^13^C]-leucine, L-[ring-^2^H_2_]-tyrosine and L-[ring-^2^H_5_]-phenylalanine tracer and indocyanine green infusions [38] and arterial and venous blood sampling. Muscle biopsy tissue samples were collected from the *M*. *vastus lateralis*.

### Protocol

All subjects were instructed to refrain from strenuous physical exercise for 3 days prior to testing. Subjects refrained from alcohol and caffeine, and consumed a standardized dinner (32.5±0.7 kJ·kg^-1^ body weight; providing 16 energy percent (en%) protein, 33 en% carbohydrate, and 51 en% fat) the evening prior to testing. The following morning, subjects arrived at the laboratory at 7:30 AM by car or public transportation. A polyethylene catheter was inserted into an antecubital vein for intravenous isotope tracer infusion. A second catheter was inserted into a dorsal hand vein of the contralateral arm and placed in a hot-box (60°C) for arterialized blood sampling to measure background indocyanine green [38], glucose, and insulin concentrations. Subsequently, the other 2 polyethylene catheters were inserted into the femoral artery and vein of the right leg for blood sampling. Ultrasound imaging was used to confirm safe insertion of the femoral lines. The arterial catheter was also used for the infusion of indocyanine green (IC-Green; Akorn, Lake forest, II). Following basal blood collection from the heated hand vein, femoral artery and femoral vein (t = -180 min), the plasma phenylalanine, tyrosine and leucine pools were primed with a single intravenous dose of 2.000, 0.615 and 3.99 μmol.kg^-1^, respectively, after which continuous tracer infusion (0.050 μmol^.1^kg.min^-1^ L-[ring-^2^H_5_]-phenylalanine, 0.015 μmol^.1^kg.min^-1^ L-[ring-^2^H_2_]-tyrosine, and 0.130 μmol^.1^kg.min^-1^ L-[1-^13^C]-leucine) was administrated using a calibrated IVAC 560 pump (San Diego, USA) until the end of the experiment. After resting in a supine position for 60 min, an arterialized blood sample from the heated hand vein, and an arterial and venous blood sample from the catheterized leg were drawn at t = -120 and -60 min. Subsequently, indocyanine green was infused in the femoral artery (1.0 mg/min) from t = -45 min until the end of the experiment (t = 300 min) with intervals as presented in Fig 2. After collecting blood samples from the heated hand vein, femoral artery, and femoral vein at t = 0 min, subjects received the test-drink containing 20 g intrinsically labeled casein protein and were asked to consume the drink within 5 min. Following the protein drink, blood samples from the heated hand vein, femoral artery, and femoral vein were collected at time points t = 20, 40, 60, 90, 120, 150, 180, 210, 240, 270 and 300 min. Muscle biopsies were obtained at t = 0, 120 and 300 min after protein ingestion, using the percutaneous needle biopsy technique [39]. Muscle samples were dissected carefully and freed from any visible non-muscle material and immediately frozen in liquid nitrogen and stored at -80°C until further analysis. After the third biopsy, all infusions were stopped, the catheters were removed, and the subjects were fed and discharged ~3 h after removal of the femoral catheters.

### Plasma analysis

Plasma glucose (Glucose HK CP, ABX Diagnostics, ref. A11A01667, Montpellier, France) concentrations were analyzed with a COBAS-FARA semi-automatic analyzer (Roche, Basel, Switzerland). Plasma insulin concentrations were determined by radio-immunoassay (Millipore, ref. HI-14K, Billerica, USA). ICG concentrations in plasma were determined spectrofotometrically as previously described [38]. For determination of plasma amino acid concentrations, 10 μL of plasma was mixed with 1500 μL 0.5 mM Tridecafluoroheptanoic acid (TDFHA) (Sigma, Zwijndrecht, The Netherlands) in water and 10 μL of the internal standard solution containing stable isotope-labeled amino acids (Cambridge Isotope Laboratories, Inc., Andover, USA) in 0.1 M HCl. Amino acid profiles were determined using ultra-performance liquid chromatography tandem mass spectrometry (UPLC-MS/MS) as described previously [40]. For measurement of plasma phenylalanine, tyrosine and leucine concentrations as well as L-[1-^13^C]-phenylalanine, L-[1-^13^C]-tyrosine, L-[ring-^2^H_5_]-phenylalanine, L-[ring-^2^H_4_]-tyrosine and L-[ring-^2^H_2_]-tyrosine enrichments, plasma amino acids were derivatized to their t-butyldimethylsilyl (TBDMS) derivatives after the addition of an internal standard. Their concentrations and ^13^C and/or ^2^H enrichments were determined by electron ionization gas chromatography-mass spectrometry (GC-MS, Agilent 6890N GC/5973N MSD Little Falls, USA) using selected ion monitoring of masses 336, 337 and 341 for unlabeled and labeled (L-[1-^13^C] and L-[ring-^2^H_5_]) phenylalanine, respectively. Masses 466, 467, 468 and 470 were assessed for unlabeled and labeled (L-[1-^13^C], L-[ring-^2^H_2_], and L-[ring-^2^H_4_]) tyrosine and masses 302 and 303 were assessed for unlabeled and labeled (L-[1-^13^C] leucine), respectively. Albumin was extracted from blood samples by adding 20% perchloric acid (PCA). Samples were centrifuged at 3500*g* at 4°C for 20 min after which the supernatant was removed. The mixed plasma protein pellet was washed and lyophilized to dryness. Amino acids were liberated by adding 6 mol L^−1^ HCl and heated at 120°C for 15 to 18 h. Thereafter, the hydrolyzed mixed plasma protein samples were processed via the same procedures as the muscle protein bound samples (described in the next paragraph). We applied standard regression curves in all isotopic enrichment analyses to assess linearity of the mass spectrometer and to control for the loss of tracer. Enrichments (MPE) were calculated according to Biolo *et al*. [41] to correct for the presence naturally occurring isotopes.

### Muscle analyses

For measurement of L-[1-^13^C]-leucine, L-[1-^13^C]-phenylalanine and L-[ring-^2^H_5_]-phenylalanine enrichment in the muscle free and muscle protein bound amino acid pool, 55 mg wet muscle tissue was freeze dried. Collagen, blood, and other non-muscle fiber materials were removed from the muscle fibers under a light microscope. The isolated muscle fiber mass was weighed and 35 volumes (7x wet weight of isolated muscle fibers x wet-to-dry ratio 5:1) of ice-cold 2% perchloric acid (PCA) was added. Thereafter, the tissue was homogenized and centrifuged, with the supernatant being collected and processed in the same manner as the plasma samples, such that intracellular free L-[1-^13^C]-leucine, L-[1-^13^C]-phenylalanine and L-[ring-^2^H_5_]-phenylalanine enrichments could be measured using their TBDMS derivatives on a GC-MS. The protein pellet was washed for 3 additional 1.5 mL washes of 2% PCA, dried, and the hydrolyzed protein fraction was dried under a nitrogen steam while heated to 120°C, then 50% acetic acid solution was added, and the hydrolyzed protein was passed over a Dowex exchange resin (AG 50W-X8, 100–200 mesh hydrogen form: Bio-Rad, Hercules, CA) using 2M NH_4_OH. The eluate was divided over 2 vials for separate measurement of L-[1-^13^C]-leucine and L-[1-^13^C]-phenylalanine (GC-C-IRMS) and L-[ring-^2^H_5_]-phenylalanine (GC-MS) as described previously [30]. In short, L-[1-^13^C]-leucine and L-[1-^13^C]-phenylalanine were derivatized to their N(O,S)-ethoxycarbonyl ethyl esters and measured using Isotope Ratio Mass Spectrometer (IRMS, Thermo Scientific, GC Isolink/Mat 253) and L-[ring-^2^H_5_]-phenylalanine became a TBDMS derivative of purified β-phenylethylamine produced by enzymatic decarboxylation, solvent extraction and was measured on the GC-MS. Standard regression curves were applied to assess linearity of the mass spectrometer and to control for the loss of tracer.

### Calculations

Whole-body amino acid kinetics (expressed in nmol phenylalanine·kg BW^-1^·min^-1^) in non-steady state conditions were calculated from the ingestion of L-[1-^13^C]-phenylalanine labeled protein with primed intravenous infusion of L-[ring-^2^H_5_]-phenylalanine and L-[ring-3,5-^2^H_2_]-tyrosine, with frequent venous and arterial blood sampling. Total, exogenous, and endogenous phenylalanine rates of appearance (Ra), as well as plasma phenylalanine availability (i.e, the fraction of dietary protein-derived phenylalanine that appeared in the systemic circulation, Phe plasma) were calculated using modified Steele’s equations [42, 43]. Furthermore, total rate of phenylalanine disappearance (Rd), utilization of phenylalanine for protein synthesis, and phenylalanine hydroxylation (first step of phenylalanine conversion to tyrosine) were calculated. Whole-body net protein balance was calculated by subtracting protein synthesis from endogenous phenylalanine appearance (Ra) and presented as the weighted mean over the 5 h (t = 0–300 min) post-prandial period.

Phenylalanine and leucine kinetics were also used to study the two-pool and three-pool models [44]. All calculations in this manuscript are based on the methodology as presented by Biolo and colleagues [23]. The two-pool model has been used by a number of research groups, thus allowing for a comparison of our results with previous results from our lab [18, 30] and data collected by others [45]. Phenylalanine was selected as the primary amino acid of interest as it is not metabolized in skeletal muscle tissue. Historically, leucine has mainly been used as the amino acid of interest [22, 41, 46] and, therefore, measurements were performed based upon the phenylalanine and/or leucine tracer where appropriate. With the two-pool model, phenylalanine enrichments and concentrations in the femoral artery and vein were used to assess amino acid uptake over the leg. These parameters are based on the extraction of the labeled phenylalanine from the femoral artery, the appearance of phenylalanine in the femoral vein, and the net arteriovenous difference of the phenylalanine concentrations [44]. Thus this model allows for the assessment of plasma phenylalanine kinetics across the leg, providing no particular insight into its intramyocellular kinetics. The three-pool model is an expansion of the two-pool model and relies not only on the measurement of the amino acid enrichments and concentrations in the femoral artery and vein, but also on the direct measurement of the amino acid enrichment in muscle free tissue water. This allows for the assessment of intracellular phenylalanine use for protein synthesis and release from protein breakdown. In addition, it is possible to calculate the rate of phenylalanine and leucine transport from the artery into the tissue and from the tissue into venous blood [23, 45]. The two- and three-pool models share the following parameters:

1 $Delivery\;to\;the\;leg=F_{in}=C_{A}\cdot BF$
2 $Output\;from\;the\;leg=F_{out}=C_{V}\cdot BF$
3 $Leg\;net\;balance\;\left(NB\right)=(C_{A}-C_{V})\cdot BF$

The other kinetic parameters of the three-pool method are calculated as follows:

4 $Total\;rate\;of\;appearance\;in\;the\;leg\;\left(total\;R_{a}\right)=(C_{A}\cdot \frac{E_{A}}{E_{V}}\;)\cdot BF$
5 $Release\;from\;the\;leg\;\left(leg\;R_{a}\right)=totalR_{a}-F_{in}=BF\cdot C_{A}\cdot \left(\left(\frac{E_{A}}{E_{V}}\right)-1\right)$
6 $Rate\;of\;disappearance\;in\;the\;leg\;\left(leg\;R_{d}\right)=totalR_{a}+NB=BF\cdot \left(\left(C_{A}-\left(\frac{E_{A}}{E_{V}}\right)\right)-C_{V}\right)$

where C_A_ and C_V_ are the plasma phenylalanine or leucine concentrations in the femoral artery and vein, respectively; E_A_ and E_V_ are phenylalanine or leucine enrichments, expressed as Mole Percentage Excess (MPE), in the femoral arterial and venous plasma, respectively; and BF is leg blood flow as calculated from the steady-state ICG concentration values in the femoral and antecubital veins, as described elsewhere [47]. Data are expressed in nmol per min per 100 mL of leg volume. The specific parameters of the three-pool model were calculated as follows:

7 $muscle\;inward\;transport=F_{M,A}=\left(\left(C_{V}\cdot \left(\frac{E_{M}-E_{V}}{E_{A}-E_{M}}\right)\right)+C_{A}\right)\cdot BF$
8 $muscle\;outward\;transport=F_{V,M}=\left(\left(C_{V}\cdot \left(\frac{E_{M}-E_{V}}{E_{A}-E_{M}}\right)\right)+C_{V}\right)\cdot BF$
9 $arteriovenous\;shunting=F_{V,A}=F_{in}-F_{M,A}$
10 $muscle\;protein\;breakdown=F_{M,O}=F_{M,A}\cdot \left(\left(\frac{E_{A}}{E_{M}}\right)-1\right)$
11 $muscle\;protein\;synthesis=F_{O,M}=F_{M,O}+NB$

Where E_M_ is phenylalanine or leucine enrichment, expressed as Mole Percentage Excess (MPE), in the muscle. Additionally, we calculated the intracellular phenylalanine and leucine availability as the sum of transport into the muscle F_M,A_ and the intracellular R_a_ from breakdown F_M,O_:

12 $intracellular\;amino\;acid\;availability=F_{M,A}+F_{M,O}$

We also calculated the fractional synthetic rate (FSR) of mixed muscle protein by measuring the incorporation rate of the phenylalanine or leucine tracer into mixed muscle protein (ΔE_P_/t) using the precursor-product model to calculate muscle protein synthesis rates:

13 $FSR=\frac{\left(\frac{\Delta E_{P}}{t}\right)}{\left(\frac{E_{M1}+E_{M2}}{2}\right)}\cdot 60\cdot 100$

Where ΔE_p_ is the change in protein-bound phenylalanine or leucine enrichment between two sequential biopsies, *t* is the time between the biopsies, and E_M(1)_ and E_M(2)_ are the phenylalanine or leucine enrichments in the muscle free or plasma pool. Data are expressed as percent per hour.

### Statistics

Twelve volunteers were included in the present experiment. All results are expressed as means ± standard error of the mean (SEM). Effect of time was calculated by repeated measures analysis, using a mixed model with unstructured covariance matrix. These calculations were performed using the procedure MIXED of SAS (version 9.3). Overall time effect and comparisons between the time-points are described. Where measurements in time are compared with those at the basal level (t = 0 min), a Dunnett test was applied, otherwise the LSD-test. In the case measurements were not repeated in time, e.g. baseline subject characteristics, a (independent) t-test was applied. Two-sided tests were applied, and statistical significance was set at *P*<0.05.

## Results

### Leg blood flow

Blood flow averaged 6.0±0.4 mL·min^-1^·100 mL leg volume^-1^ in the basal state and did not change during the early (6.2±0.5 mL·min^-1^·100 mL leg volume^-1^) or late (5.6±0.3 mL·min^-1^·100 mL leg volume^-1^) post-prandial phase when compared to basal values (*P* = 0.92 and *P* = 0.10, respectively). Median blood flow during the entire post-prandial phase averaged 5.8±0.4 mL·min^-1^·100 mL leg volume^-1^ and was applied for all calculations.

### Plasma glucose and insulin concentrations

Fasting plasma glucose and insulin levels averaged 4.83±0.10 mmol·L^-1^ and 10.2±0.7 mU·mL^-1^, respectively. Following protein ingestion, glucose concentrations significantly increased during the early post-prandial phase (t = 0–120 min; 5.06±0.08 mmol·L^-1^ (*P* = 0.014)) and returned to baseline values (t = 120–300 min; 4.83±0.07 mmol·L^-1^ (*P* = 0.992)) during the late stages of the 5 h post-prandial period. Plasma insulin concentrations increased upon protein ingestion in the early post-prandial phase (13.1±1.2 mU·L^-1^; *P*<0.01) and declined over time with average values of 8.5±0.8 mU·L^-1^ observed during the late post-prandial phase (*P* = 0.014).

### Plasma amino acid concentrations

**Fig 3:**
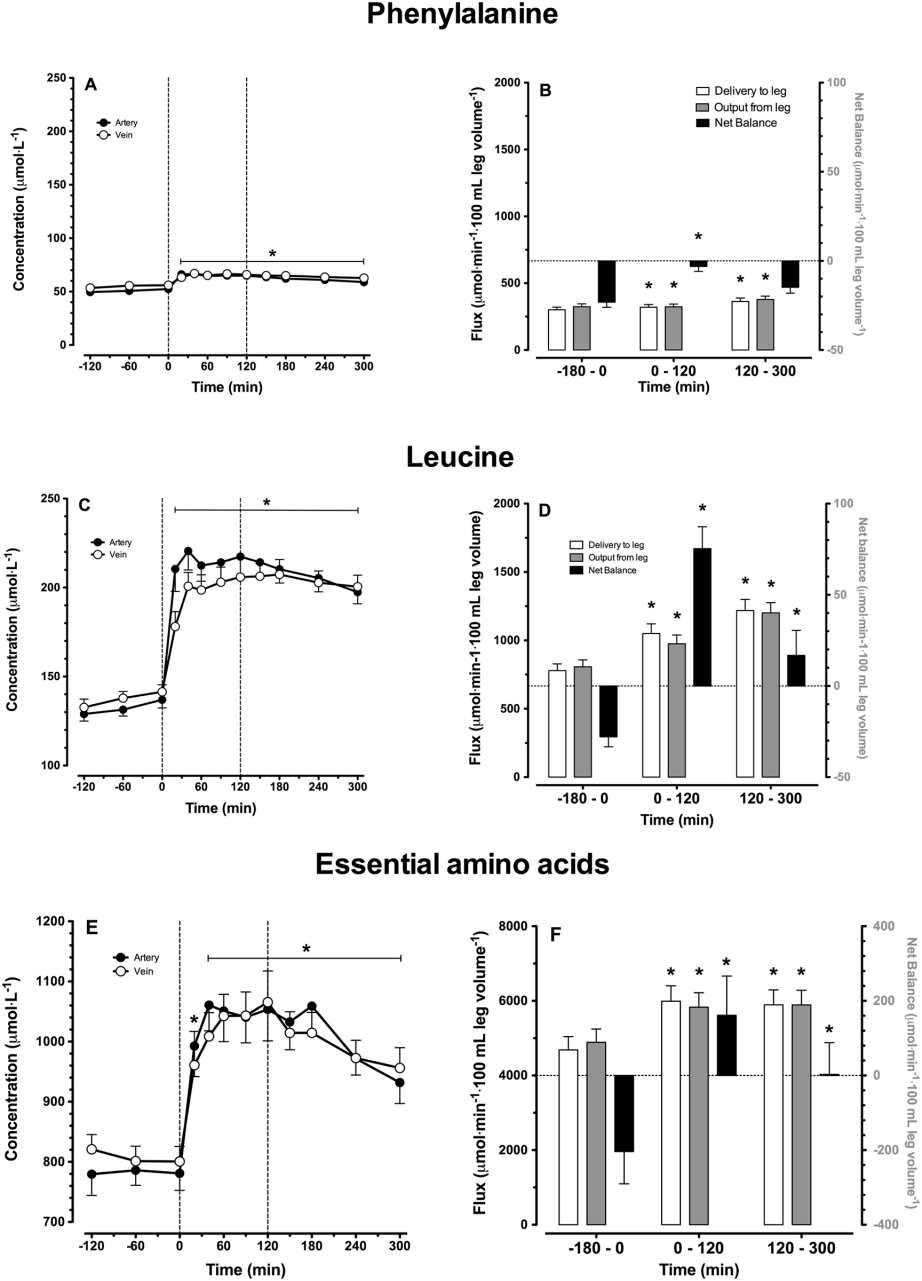
Amino acid concentrations and leg net balances. Arterial and venous plasma amino acid concentrations (A, C, E) and delivery, output and net balance over the leg (B, D, F). Values are expressed as means ± SEM. * Indicates a significant difference compared to baseline level (t = 0 min), *P*<0.05.

Arterial and venous plasma phenylalanine, leucine, and essential amino acid (EAA) concentrations over time are shown in Fig 3A, 3C and 3E, respectively. After ingestion of the intrinsically labeled casein protein, plasma amino acid concentrations increased rapidly and remained significantly elevated compared to basal values during the entire post-prandial phase (*P*<0.001). Peak arterial plasma phenylalanine, leucine and EAA concentrations averaged 66±2, 220±11 and 1112±77 μmol·L^-1^, respectively. Peak venous plasma phenylalanine and leucine concentrations averaged 67±2, 207±8 and 1080±50 μmol·L^-1^, respectively. Phenylalanine and leucine delivery to the leg, output from the leg, as well as leg net balance are presented in Fig 3B, 3D and 3F, respectively. Phenylalanine, leucine and total EAA balance increased following protein ingestion, becoming positive during the early and/or late stages of the post-prandial phase. All changes are significant compared to basal values, except for phenylalanine net balance during the later stages of the post-prandial phase (*P* = 0.226).

### Plasma amino acid enrichments

**Fig 4:**
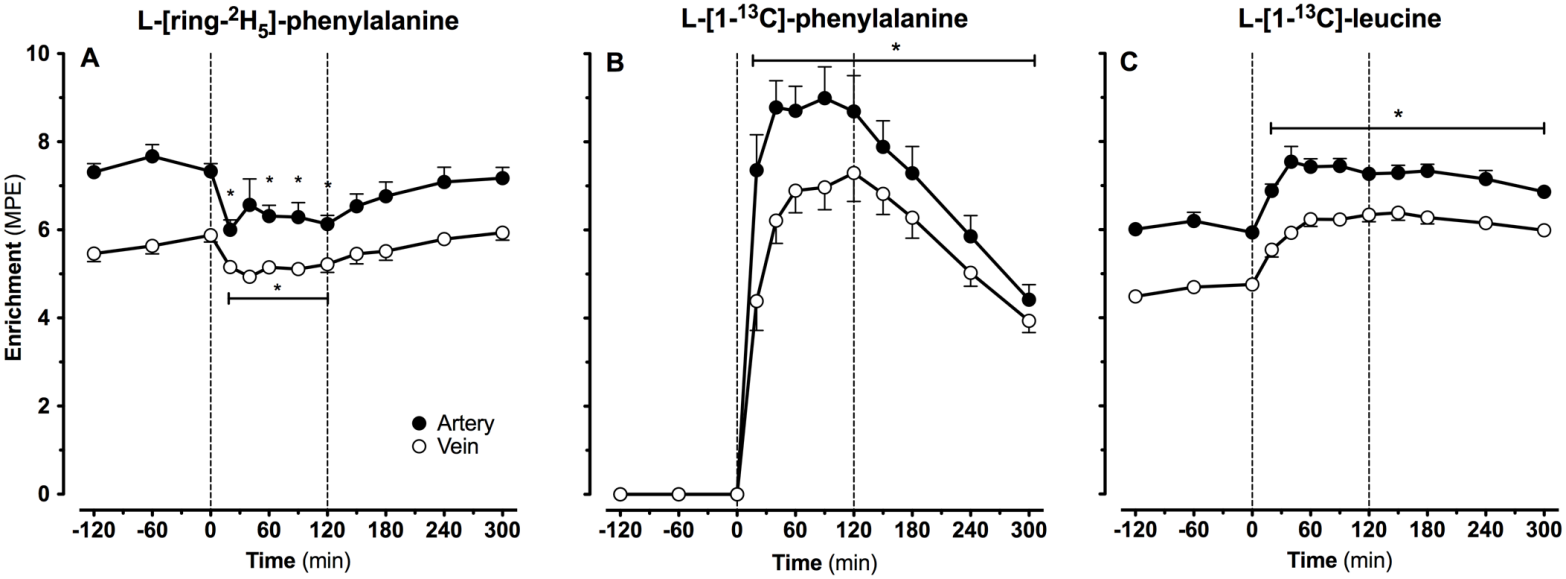
Amino acid enrichments. Arterial and venous plasma L-[ring-^2^H_5_]-phenylalanine (A), L-[1-^13^C]-phenylalanine (B) and L-[1-^13^C]-leucine (C) enrichments. Intrinsically labeled protein was ingested at t = 0 min. Values are expressed as means ± SEM. * Indicates a significant difference compared to baseline level (t = 0 min), *P*<0.05.

Continuous infusions with L-[ring-^2^H_5_]-phenylalanine and L-[1-^13^C]-leucine were applied from t = -180 until t = 300 min. Intrinsically L-[1-^13^C]-phenylalanine and L-[1-^13^C]-leucine labeled casein was ingested at t = 0 min. Plasma tracer enrichments from the infused and/or ingested amino acid tracers sampled from the femoral artery and vein are presented in Fig 4A–4C. Basal arterial and venous L-[ring-^2^H_5_]-phenylalanine enrichments averaged 7.43±0.19 and 5.67±0.15 MPE, respectively. Basal arterial and venous L-[1-^13^C]-leucine enrichments averaged 6.05±0.12 and 4.64±0.10 MPE, respectively. Average arterial L-[ring-^2^H_5_]-phenylalanine enrichments during the early and late post-prandial phase were 11±2 and 3±2% lower than enrichments observed in the basal state (*P*<0.001 and *P* = 0.18, respectively). After protein ingestion arterial and venous plasma L-[1-^13^C]-phenylalanine enrichments increased rapidly, reaching peak levels of 8.99±0.71 and 7.30±0.65 MPE after 90 and 120 min, respectively (Fig 4B). L-[1-^13^C]-leucine entered the circulation via intravenous infusion and followed digestion and amino acid absorption of the ingested L-[1-^13^C]-phenylalanine and L-[1-^13^C]-leucine labeled protein. After ingestion, small but significant increases in plasma L-[1-^13^C]-leucine enrichments were observed during the early (+0.47±0.07 MPE) and late stages (+1.13±0.13 MPE) of the post-prandial period (both *P*<0.001). Peak arterial and venous plasma L-[1-^13^C]-leucine enrichments were observed 90 and 150 min after protein ingestion and averaged 7.44±0.17 and 6.38±0.17 MPE, respectively (Fig 4C).

### Plasma amino acids kinetics

**Fig 5:**
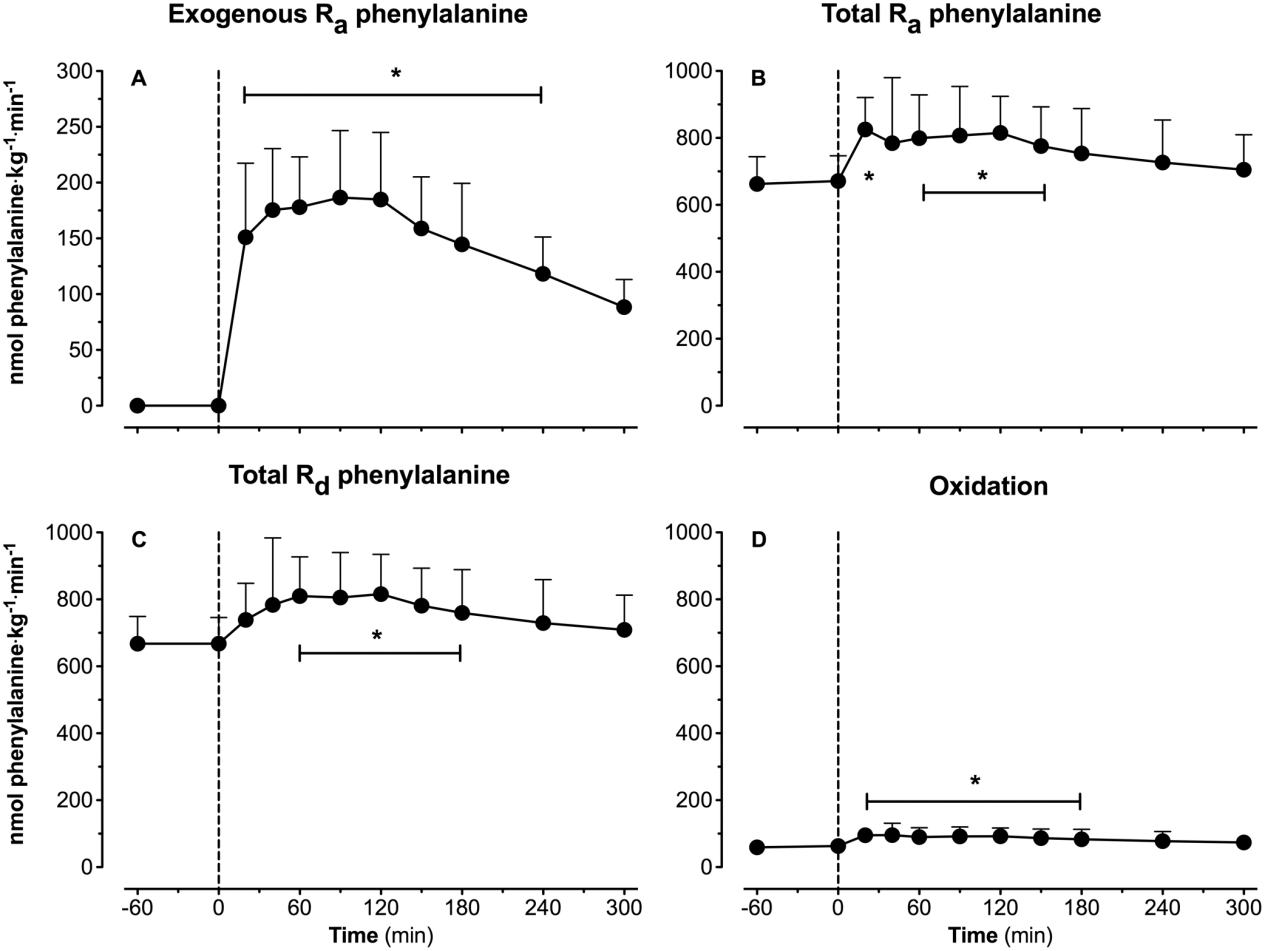
Plasma amino acids kinetics. Exogenous phenylalanine rate of appearance (R_a_) (A), total phenylalanine R_a_ (B), total phenylalanine rate of disappearance (R_d_) (C) and phenylalanine-to-tyrosine conversion (oxidation) (D). Values represent means ± SEM. * Indicates a significant difference compared to baseline level (t = 0 min), *P*<0.05.

Whole body plasma amino acid kinetics are presented in Fig 5A–5D. Immediately after protein ingestion, exogenous L-[1-^13^C]-phenylalanine started to appear in the circulation. Exogenous phenylalanine appearance rates reached peak values at 90 min after protein ingestion (187±17 ηmol·kg^-1^·min^-1^; Fig 5A). Whole body plasma phenylalanine disappearance rates followed the rates of appearance and showed significant post-prandial phenylalanine disposal (Fig 5B and 5C). Conversion of L-[ring-^2^H_5_]-phenylalanine to L-[ring-^2^H_4_]-tyrosine is shown in Fig 5D and averaged 62±3 ηmol·kg^-1^·min^-1^ during the basal state, and increased to 79±5 ηmol·kg^-1^·min^-1^ during the post-prandial phase (*P* = 0.0037). In this experiment, post-prandial L-[1-^13^C]-phenylalanine retention in the splanchnic area averaged 44.7±2.7%, with thus 55.3±2.7% of the ingested L-[1-^13^C]-phenylalanine appearing in the circulation during the 5 h post-prandial period.

### Whole-body amino acid kinetics

**Fig 6:**
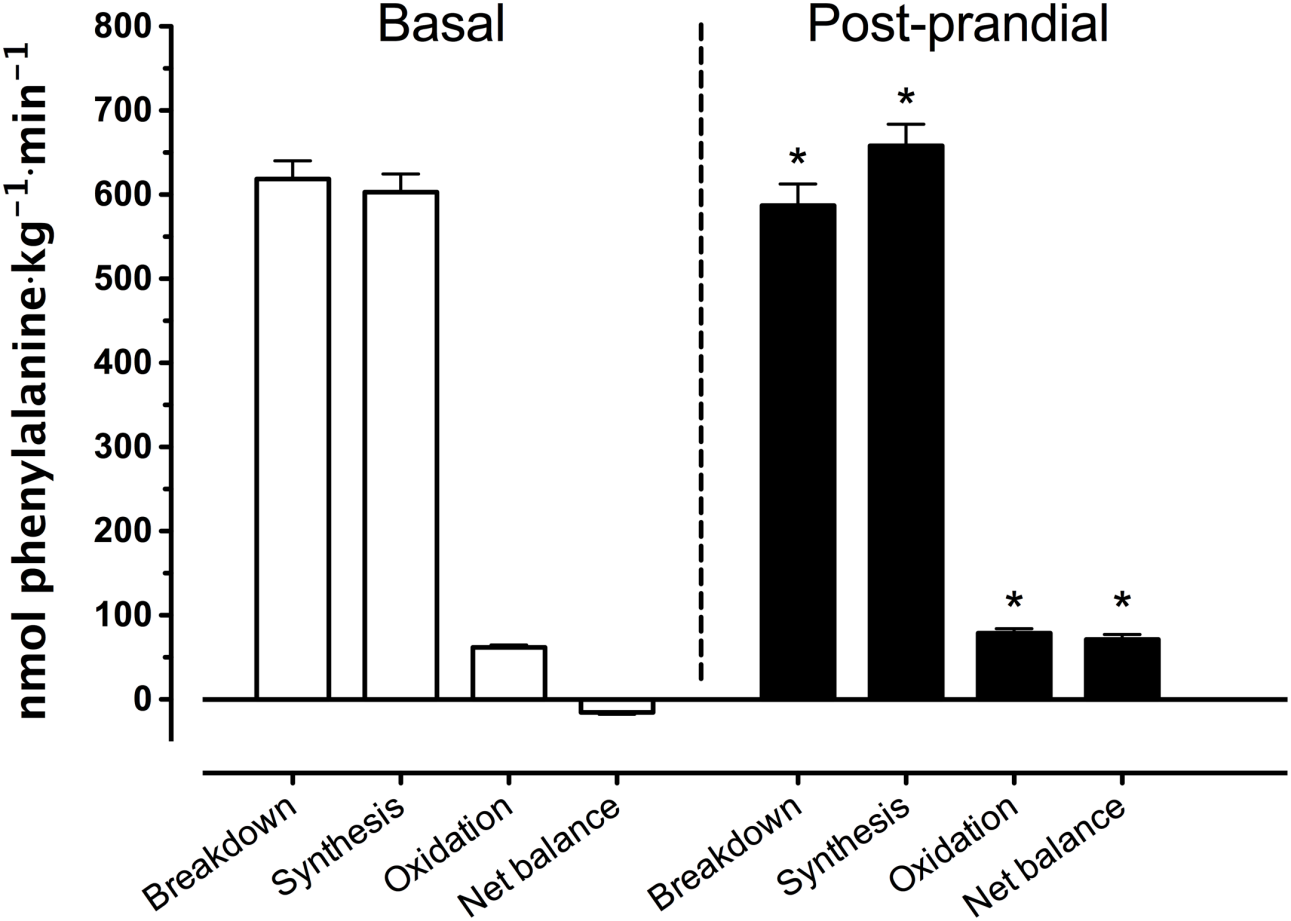
Whole body phenylalanine protein kinetics. Rates of whole body protein breakdown, synthesis, and oxidation and whole-body net protein balance (expressed as nmol phenylalanine·kg^-1^·min^-1^) assessed during the basal (t = -180 –t = 0 min) and post-prandial (t = 0 –t = 300 min) period. Values represent means ± SEM. * Indicates a significant difference compared to basal values, *P*<0.05.

Whole-body protein breakdown, synthesis, oxidation rates, and net balance are presented in Fig 6. During the basal state, breakdown rates exceeded synthesis rates, resulting in a negative net protein balance. In the post-prandial state, net protein balance became positive compared to basal values due to a decline in whole-body protein breakdown rates from 619±22 to 587±26 nmol phenylalanine·kg^-1^·min^-1^ and an increase in whole-body protein synthesis rates from 603±22 to 658±25 nmol phenylalanine·kg^-1^·min^-1^.

### 3-pool kinetics

Results of the 3-pool model calculations during de basal and post-prandial period are presented in Table 2. Fluxes (expressed in ηmol·min^-1^·100 mL leg volume^-1^) increased during the post-prandial phase, compared to basal values. Exceptions were observed for leucine with respect to shunting (F_V,A_) and IC-availability. No significant changes were detected for phenylalanine rate of intracellular amino acid appearance from endogenous sources (F_M,O_) and the rate of utilization of intracellular amino acids, i.e. protein synthesis and other fates (F_O,M_).

**Table 2:** Leg phenylalanine and leucine kinetics.

	Phenylalanine (*n* = 12) (nmol·min^-1^·100 mL leg volume^-1^)			Leucine (*n* = 12) (nmol·min^-1^·100 mL leg volume^-1^)		
Flux	Basal	Post-prandial	*P*	Basal	Post-prandial	*P*
**Total leg R**_**a**_	391±27	421±30	*<0*.*01*	1015±60	1369±85	*<0*.*001*
**Leg R**_**a**_	93±9	72±8	*<0*.*01*	235±23	196±22	*<0*.*01*
**Leg R**_**d**_	368±26	411±31	*<0*.*01*	987±59	1409±92	*<0*.*001*
**F**_**M,A**_	302±20	353±26	*<0*.*001*	744±47	1062±78	*<0*.*001*
**F**_**V,M**_	326±21	365±25	*<0*.*01*	773±48	1023±70	*<0*.*001*
**F**_**V,A**_	6±8	0±8	*n*.*s*.	56±8	102±10	*<0*.*001*
**F**_**M,O**_	161±29	101±19	*<0*.*01*	524±65	541±63	*n*.*s*.
**F**_**O,M**_	137±27	89±20	*<0*.*01*	495±65	580±68	*n*.*s*.
**IC availability**	464±39	454±34	*n*.*s*.	1268±101	1603±127	*<0*.*001*
**Net balance**	-24±3	-11±2	*<0*.*01*	-28±5	40±11	*<0*.*001*

Values are expressed as means ± SEM. Phenylalanine and leucine kinetics (ηmol·min^-1^·100 mL leg volume^-1^) expressed as (total) leg R_a_, leg R_d_, transport into (F_M,A_) and from the muscle (F_V,M_), a–v shunting (F_V,A_), release from proteolysis (F_M,0_), utilization for protein synthesis (phenylalanine) and synthesis and oxidation (leucine) (F_0,M_), and intracellular availability (IC availability) before protein ingestion (Basal) and during the entire post-prandial period (t = 0 min–t = 300 min).

### Muscle free tissue and muscle enrichments

Muscle biopsies were obtained at t = 0, 120 and 300 min. Plasma albumin obtained prior to tracer infusion was used to determine baseline L-[ring-^2^H_5_]-phenylalanine enrichment to allow calculation of basal muscle protein synthesis rates using the single biopsy approach as validated previously for L-[ring-^2^H_5_]-phenylalanine [48]. Muscle-free and muscle protein bound tissue enrichments are summarized in Table 3.

**Table 3:** Muscle tissue-free and muscle protein bound enrichments.

Sample (*n* = 12)	Stable isotope tracer	-180 min	0 min	120 min	*P*	300 min	*P (* * *)*	*P (* ^#^ *)*
**Muscle free** (MPE)	**L-[ring-** ^**2**^ **H**_**5**_ **]-phenylalanine**	0	4.937±0.228	5.136±0.285	*1*.*000*	5.224±0.229	*0*.*854*	*1*.*000*
	**L-[1-** ^**13**^ **C]-phenylalanine**		0	5.658±0.585*	*<0*.*001*	3.310±0.296*	*<0*.*001*	*0*.*104*
	**L-[1-** ^**13**^ **C]-leucine**		0	5.372±0.232*	*<0*.*001*	4.831±0.220*	*<0*.*001*	*0*.*162*
**Muscle protein bound** (MPE)	**L-[ring-** ^**2**^ **H**_**5**_ **]-phenylalanine**	0	0.0066±0.0005	0.0127±0.0007*	*<0*.*001*	0.0213±0.0013* ^#^	*<0*.*001*	*<0*.*001*
	**L-[1-** ^**13**^ **C]-phenylalanine**		0	0.0060±0.0017*	*<0*.*01*	0.0201±0.0025* ^#^	*<0*.*001*	*<0*.*001*
	**L-[1-** ^**13**^ **C]-leucine**		0	0.0112±0.0018*	*<0*.*01*	0.0204±0.0017* ^#^	*<0*.*01*	*<0*.*001*

Muscle tissue-free and muscle protein bound enrichments at the start of the experiment (t = -180 min), the end of the basal period (t = 0 min), the early (t = 120 min) and the late (t = 300 min) stages of the post-prandial phase (MPE). Muscle free and muscle protein bound L-[ring-^2^H_5_]-phenylalanine enrichments at t = -180 min were assessed based upon plasma albumin as an alternative of a baseline biopsy sample as described previously [48]. Values are expressed as means ± SEM.

*: Statistically different from basal (t = 0 min).

^#^: Statistically different from the early stage of the post-prandial state (t = 120 min).

Data were analyzed by repeated measures ANOVA.

Muscle protein bound L-[ring-^2^H_5_]-phenylalanine enrichments significantly increased over time reaching highest levels at t = 300 min (*P*<0.001). No significant increases were observed in muscle free L-[ring-^2^H_5_]-phenylalanine enrichments.

Upon ingestion of the intrinsically L-[1-^13^C]-phenylalanine (and L-[1-^13^C]-leucine) labeled protein, muscle protein bound L-[1-^13^C]-phenylalanine enrichments increased in biopsy samples collected 2 and 5 h after protein ingestion (*P*<0.001). The increase in muscle free L-[1-^13^C]-phenylalanine enrichments over time did not reach statistical significance (*P* = 0.104).

The appearance of L-[1-^13^C]-leucine in the circulation from the intravenous L-[1-^13^C]-leucine infusion plus the digestion and amino acid absorption of intrinsically L-[1-^13^C]-leucine (and [1-^13^C]-phenylalanine) labeled protein led to significant increases in muscle protein bound L-[1-^13^C]-leucine enrichments at 2 and 5 h after protein ingestion (*P*<0.001). The increase in muscle free L-[1-^13^C]-leucine enrichment over time did not reach statistical significance (*P* = 0.162).

### Mixed muscle FSR

**Fig 7:**
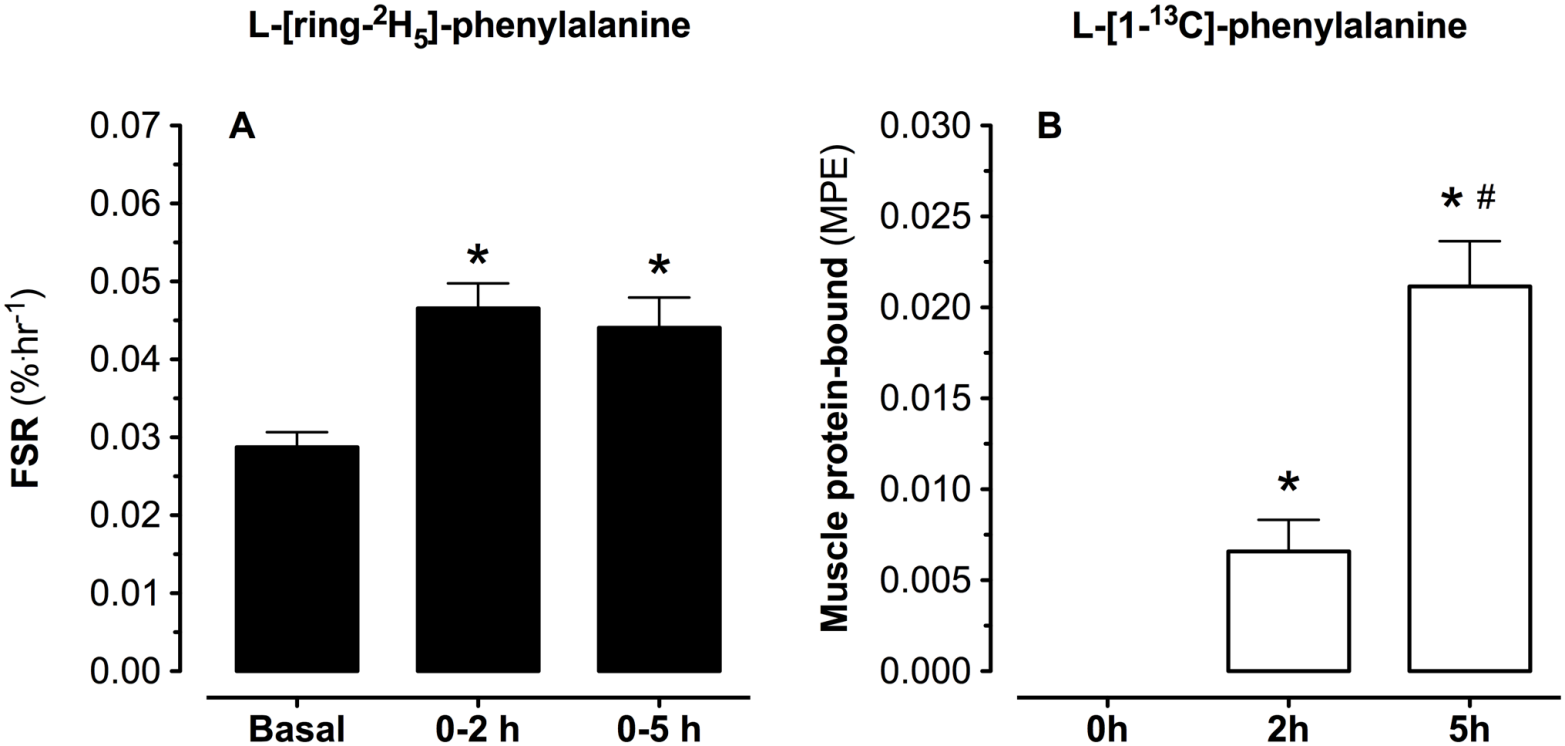
Mixed muscle protein synthesis and muscle tissue enrichments. Muscle protein synthesis rates (FSR) based on plasma L-[ring-^2^H_5_]-phenylalanine (A), and L-[1-^13^C]-phenylalanine (B) protein bound muscle protein enrichments (MPE). Values represent means ± SEM. * Indicates a significant difference compared to basal values, *P*<0.05. ^#^ Indicates a significant difference compared to basal values, *P*<0.05.

L-[ring-^2^H_5_]-phenylalanine based mixed muscle protein fractional synthesis rates, and L-[1-^13^C]-phenylalanine muscle tissue enrichments (MPE) are presented in Fig 7. Mixed-muscle protein synthesis rates, with the weighed mean plasma L-[ring-^2^H_5_]-phenylalanine enrichment as precursor, averaged 0.029±0.002%·h^-1^ in the basal, overnight fasted state. During the early digestion phase (0–2 h), and over the entire post-prandial phase (0–5 h), mixed muscle protein synthesis rates increased to 0.047±0.003 and 0.044±0.004%·h^-1^, respectively (both *P*<0.01 compared to the basal state). The mean ± SEM (95% CI) of the differences between basal and post-prandial muscle protein synthesis rates averaged 0.0161±0.0031 (0.0110–0.0226) with an effect size of 1.79.

Post-prandial muscle protein synthesis rates were also calculated based upon the L-[1-^13^C]-leucine tracer resulting in muscle protein synthesis rates averaging 0.063±0.014 and 0.058±0.006%·h^-1^ during the early (0–2 h) and the complete 5 h post-prandial period. Fractional synthesis rates were calculated with the weighed average plasma L-[ring-^2^H_5_]-phenylalanine or L-[1-^13^C]-leucine enrichments being used as precursor pool. Muscle protein synthesis rates calculated based upon the muscle free precursor pool(s) resulted in similar findings (*data not shown*). Mixed muscle protein L-[1-^13^C]-phenylalanine enrichments increased from 0 to 0.0060±0.0017 and 0.0201±0.0025 MPE at 2 and 5 h after protein ingestion, respectively, showing substantial incorporation of dietary protein derived L-[1-^13^C]-phenylalanine into *de novo* muscle protein (*P*<0.001).

## Discussion

The present study demonstrates that ingestion of a single meal-like amount of protein allows ~55% of these protein-derived amino acids to become available in the systemic circulation during a 5 h post-prandial period. The protein-derived amino acids are taken up in skeletal muscle tissue, improving leg and whole-body protein balance, and increasing muscle protein synthesis rates beyond basal, post-absorptive values. Following ingestion of 20 g casein, ~11% of the dietary protein derived amino acids (2.2±0.3 g) were incorporated in *de novo* muscle protein within the 5 h post-prandial period.

In the present study we combined ingestion of specifically produced intrinsically L-[1-^13^C]-phenylalanine labeled protein with continuous intravenous infusion of L-[ring-^2^H_5_]-phenylalanine to allow assessment of dietary protein digestion and absorption kinetics *in vivo* in humans. Ingestion of 20 g casein was followed by proper digestion and amino acid absorption, as indicated by the rapid increase in both plasma phenylalanine, and total essential amino acid concentrations (Fig 3) as well as plasma L-[1-^13^C]-phenylalanine enrichments (Fig 4). Following the ingestion of 20 g protein, 25±2% of the dietary protein derived phenylalanine had been released into the circulation within the first 2 h of the post-prandial phase, followed by another 30±2% during the subsequent 3 h. These findings are in line with previous observations using intrinsically labeled protein under various conditions in different (sub)populations [5, 49, 50] and with earlier estimations of splanchnic amino acid extraction during continuous oral administration of free crystalline phenylalanine in healthy, older men [51]. The present data show that 55±3% of dietary protein derived amino acids are being released in the circulation during a 5 h post-prandial period, making these amino acids available as precursors for *de novo* muscle protein synthesis. The other dietary protein derived amino acids (~45%) are retained in the splanchnic area, providing precursors for gut and liver protein synthesis to help sustain their functional mass [52].

The dietary protein derived amino acids appearing in the circulation were effectively taken-up by skeletal muscle tissue. Using an AV model we measured amino acid inflow and outflow from the leg, allowing us to determine net amino acid uptake over the leg (Fig 3B, 3D and 3F). Protein ingestion did not result in a measurable increase in leg blood flow, as has been reported previously following amino acid [51, 53] or protein [54–56] administration. Therefore, differences in amino acid uptake over the leg were fully attributed to the differences between arterial and venous amino acid concentrations (Fig 3A, 3C and 3E). Leg protein balance was negative in the basal, post-absorptive state and improved substantially following protein ingestion. Phenylalanine net balance improved following protein ingestion but remained slightly negative, which is commonly observed and attributed to the release of phenylalanine from subdermal and intramuscular fat depots [57]. In agreement, a positive net amino acid balance was observed following protein ingestion when arteriovenous differences in leucine or the sum of all essential amino acid concentrations were assessed (Fig 3). The essential amino acid net balance increased from -204±87 μmol·min^-1^·100 mL leg volume^-1^ in the basal post-absorptive state to 66±77 μmol·min^-1^·100 mL leg volume^-1^ following protein ingestion.

A more positive protein balance was not only observed in the leg but also on a whole-body level (Fig 6) The post-prandial increase in plasma amino acid availability stimulated whole body protein synthesis and oxidation rates, and inhibited proteolysis, resulting in a positive whole-body protein balance. However, whole-body protein kinetics do not necessarily reflect changes in muscle protein synthesis or breakdown rates in skeletal muscle tissue as they do not differentiate between tissues and do not provide information on the metabolic fate of the amino acids taken up in these tissues [58]. To provide more insight in the impact of protein ingestion on post-prandial amino acid handling in skeletal muscle tissue and to assess not only amino acid uptake and release from the leg, but also amino acid uptake and release from skeletal muscle tissue, we also applied a three-pool model [23]. The post-prandial rise in plasma amino acid availability stimulated amino acid uptake in skeletal muscle tissue thereby increasing intramyocellular amino acid availability (Table 3). The latter was accompanied by a reduction in muscle protein breakdown, F_m,o_, (for phenylalanine but not for leucine) but without measurable increases in muscle protein synthesis, F_o,m_, in the post-prandial compared with the post-absorptive state (for both phenylalanine and leucine). Of course, the two- and three-pool models have their limitations, with methodological complications associated with assessing skeletal muscle blood flow (or tissue perfusion). Nonetheless, these data provide us more insight in the greater extraction of amino acids over the leg in the post-prandial when compared with post-absorptive state.

The three-pool model was used to provide us with estimations of intramyocellular trafficking of the amino acids taken up in the leg [23], but this model does not provide a direct evaluation of the impact of protein ingestion of muscle protein synthesis rates or the metabolic fate of the ingested protein derived amino acids towards *de novo* muscle protein accretion. Therefore, we measured both muscle free and muscle protein bound L-[ring-^2^H_5_]-phenylalanine, L-[1-^13^C]-leucine and L-[1-^13^C]-phenylalanine enrichments in muscle samples collected prior to protein ingestion and 2 and 5 h into the post-prandial phase (Table 3). By also measuring plasma albumin protein bound [ring-^2^H_5_]-phenylalanine enrichments from a blood sample collected prior to [ring-^2^H_5_]-phenylalanine infusion, we were able to assess basal muscle protein synthesis rates (between t = -180–0 min) using the single biopsy methodology that has been validated previously for FSR measurements based on prolonged [ring-^2^H_5_]-phenylalanine infusion [48, 59, 60]. Ingestion of 20 g protein increased muscle protein synthesis rates by 58±11% from 0.029±0.002%·h^-1^ in the post-absorptive state to 0.047±0.003%·h^-1^ and 0.044±0.004%·h^-1^ during the early (2 h) and the entire (5 h) post-prandial phase (*P*<0.01: Fig 7A). Postprandial muscle protein synthesis rates based upon L-[1-^13^C]-leucine infusion averaged 0.063±0.014%·h^-1^ and 0.058±0.006%·h^-1^ calculated during the early and over the entire 5 h post-prandial phase, respectively.

In addition to the assessment of basal and post-prandial muscle protein synthesis following primed continuous infusion of L-[ring-^2^H_5_]-phenylalanine or primed continuous infusion of L-[1-^13^C]-leucine combined with the ingestion of intrinsically L-[1-^13^C]-leucine labeled protein, we also evaluated the metabolic fate of the ingested protein by measuring the incorporation of dietary protein derived L-[1-^13^C]-phenylalanine into the muscle protein pool. Based upon the pioneering work of Beaufrere and Boirie [43, 46, 61, 62], we have further developed the production of intrinsically L-[1-^13^C]-phenylalanine labeled milk protein with phenylalanine enrichment levels exceeding 30–35 MPE by infusing large amounts of L-[1-^13^C]-phenylalanine in lactating Holstein cows [21, 32, 63]. Application of this protein in human nutrition studies does not only allow us to assess *in vivo* dietary protein digestion and absorption kinetics but also to directly measure the incorporation of dietary protein derived L-[1-^13^C]-phenylalanine into muscle protein *in vivo* in human. Ingestion of the intrinsically labeled protein resulted in large increases in muscle free as well as muscle protein bound L-[1-^13^C]-phenylalanine enrichments (Table 3). The latter proving the uptake and subsequent use of the dietary protein derived amino acids for *de novo* muscle protein synthesis during the early and late post-prandial stage. The use of intrinsically L-[1-^13^C]-phenylalanine labeled protein with an enrichment level exceeding 35 MPE allows us to assess the metabolic fate of the ingested dietary protein bound phenylalanine by measuring the increase in skeletal muscle protein bound L-[1-^13^C]-phenylalanine enrichments 2 and 5 hours after protein ingestion (Fig 7B). These values do not represent fractional muscle protein synthesis rates but provide insight in the post-prandial incorporation of dietary protein derived amino acids into *de novo* muscle protein. The increase in L-[1-^13^C]-phenylalanine enrichments during the post-prandial period correlated well with the post-prandial FSR values calculated for both L-[ring-^2^H_5_]-phenylalanine (R^2^ = 0.38; *P*<0.06) and L-[1-^13^C]-leucine (R^2^ = 0.37; *P*<0.07). Assuming that all appendicular muscle mass has obtained a similar L-[1-^13^C]-phenylalanine enrichment as observed in the *M*. *vastus lateralis* biopsy sample of each individual, a total of 0.040±0.006 g L-[1-^13^C]-phenylalanine has been incorporated in the appendicular skeletal muscle mass throughout the 5 h post-prandial period. This represents 11.0±1.7% of the L-[1-^13^C]-phenylalanine that was provided within the 20 g intrinsically L-[1-^13^C]-phenylalanine labeled casein. Consequently, we confirm our hypothesis that following the ingestion of a single meal-like bolus of protein as much as ~11% of the protein derived amino acids (2.2±0.3 g) are directly taken up in skeletal muscle tissue and used as amino acid precursors to support *de novo* muscle protein synthesis during a 5 h postprandial period.

The present study provides a complete and comprehensive insight in the metabolic fate of dietary protein derived amino acids following the ingestion of a single meal-like bolus of protein. Though it has been well established that protein intake increases muscle protein synthesis rates [54–56], these data show that a substantial part of the ingested protein derived amino acids are taken up and directly used to support the post-prandial rise in muscle protein synthesis rate following ingestion of a single bolus of protein.. The dietary protein derived amino acids not only present themselves as strong signaling molecules, but also act as direct precursors for *de novo* muscle protein synthesis. In short, ingestion of a single meal-like amount of protein allows ~55% of the protein derived amino acids to become available in the circulation, thereby improving whole-body as well as leg muscle protein balance. Approximately 20% of the protein derived plasma amino acids will be taken up in skeletal muscle tissue during a 5 h post-prandial period, thereby stimulating muscle protein synthesis rates and providing precursors for *de novo* muscle protein. In conclusion, *you are what you just ate*.
